# Mass Spectrometry-Based Method for Multiple Allergens Control: Application to Bakery Goods

**DOI:** 10.3390/foods14050726

**Published:** 2025-02-20

**Authors:** Anna Luparelli, Elisabetta De Angelis, Rosa Pilolli, Francesca Lambertini, Michele Suman, Linda Monaci

**Affiliations:** 1 Institute of Sciences of Food Production, National Research Council (ISPA-CNR), Via G. Amendola, 122/O, 70126 Bari, Italy; anna.luparelli@ispa.cnr.it (A.L.); elisabetta.deangelis@ispa.cnr.it (E.D.A.); rosa.pilolli@ispa.cnr.it (R.P.); 2 Barilla G. R. F.lli SpA, Analytical Food Science Research, Via Mantova 166, 43122 Parma, Italy; francesca.lambertini@barilla.com (F.L.); michele.suman@barilla.com (M.S.)

**Keywords:** high resolution mass spectrometry, multi-target methods, food allergens, hidden allergens, food processing, bakery products

## Abstract

In recent years, mass spectrometry has played a key role as a confirmatory method to unequivocally identify multiple allergens, increasing the level of protection of allergic consumers. Despite advances made in methods of development, food processing still represents a critical issue in terms of the detection and accurate quantification of allergens due to chemical/structural modifications that can occur on the protein moiety or interferences of matrix compounds that might impair their final detection. Based on the multi-allergen MS/MS method devised within the ThrAll project, in this paper, we investigated the applicability of the developed method for the detection of traces of allergenic ingredients including egg, milk, soy, almond, hazelnut, peanuts, and sesame in two different kind of food matrices, namely cookies and rusks. The products were produced at laboratory scale in a food pilot plant that underwent different technological and thermal treatments. The challenge was to validate, in these extensively processed foods, the selected proteotypic peptide-markers capable of tracing the culprit ingredients in baked goods despite the processing the foods had undergone for their production. To accomplish this goal, the multi-target method developed on a low-resolution MS platform was transferred to a high-resolution MS system, and the pre-identified markers were also checked and validated on the new platform in order to be considered robust markers able to be indistinctly used on both types of platforms. Finally, the sensitivity of the method in terms of the Limit of Detection (LOD) and Limit of Quantification (LOQ) was calculated and the effect of the processing conditions on allergens detection in both baked goods was also investigated.

## 1. Introduction

Currently, there is no cure for food allergies, and sufferers can only rely on the correct labelling of foods to avoid experiencing undesired reactions [1]. To safeguard the health of consumers and help them in choosing safe foods, EU legislation (Regulation EU No 1169/2011) [2] mandated the labelling of allergenic ingredients, or their derivatives belonging to 14 allergens classes, whenever used as ingredients in the formulation of a product, requiring this to be clearly emphasized on the food packaging. This indication is a very useful tool for allergic patients; however, incorrectly labelled food is on the rise and does not always adequately contain information about the allergens present in foods [3,4], which poses a safety risk to allergic patients, with food-induced anaphylaxis being amongst the most hazardous ones [5].

On the other hand, a non-negligible risk factor is represented by novel foods [6] or by the presence of allergenic traces resulting from unpredictable contamination such as cross-contamination occurring in industrial plants. According to a recent study covering the years 2008–2018, a total of 2932 incidents and recalls due to food safety reasons were reported, 46% of which (1354 incidents) were due to undeclared food allergens presence [7]. Cross-contamination due to residues of allergenic ingredients contaminating foods expected to be allergens-free, can occur at different levels along the food chain: in the raw materials, during the storage, or along the pipeline for its production by sharing the same equipment with which allergenic foods are also processed. As is well documented, various food products, among which snacks and baked foods are those most likely contaminated, might be cross-contaminated by a restricted number of allergens that represent the targets of our study, namely milk [8,9], peanuts [10], soy [11,12], egg [9,13], nuts (almond and hazelnut) [8], and sesame [14,15,16].

To protect allergic consumers from inadvertently contaminating allergens, companies resort to the use of Precautionary Allergen Labelling (PAL) as a voluntary and non-mandatory action.

Since PAL is not mandatory and, to date, there are no clear rules about its use, the absence of PAL on food packaging does not mean that consumers can be safe in ingesting that food and neither does the presence of the PAL really mean that the suspected food does contain the culprit allergens [17]. Efforts in PAL harmonization and in the correct use of PAL across food industries is now a priority of many expert groups worldwide such as the WAO Food Allergy Committee, the ILSI expert panel on PAL, and FAO/WHO [18].

In this regard, having at our disposal sensitive and accurate analytical methods capable of quantifying tiny amounts of several allergens that might simultaneously contaminate foods is crucial and may also be essential for PAL implementation in order to protect allergic consumers.

Food processing represents another issue in the development of robust and reliable methods for allergens quantification due to the numerous and different chemical/structure modifications that could occur at the protein level and the interfering compounds that might alter the outcome of the analysis [19,20,21,22,23], impairing detection of the selected peptide-markers.

In the present study, we assessed the applicability of the LC-MS method optimized and developed in a previous project to the simultaneous detection and quantification of residuals amounts of eggs, milk, soy, almond, hazelnut, peanuts, and sesame in two different types of bakery products—cookies and rusks—produced at a laboratory scale, products that underwent different technological and thermal treatments.

A brief description of the production process for cookies and rusks is provided in order to highlight differences in the complexity of the unit operations, in particular referring to the heating conditions and the presence/absence of a fermentation step. Specifically, rusk was chosen as a highly processed food, as its production involves more intensive technological phases compared to those used for producing other bakery goods.

Cookies and rusks were produced in three different formulations: allergen-free and incurred with allergens at two nominal concentration levels, 24 and 48 μg of Total Allergenic Food Protein per g of Food (hereafter referred to as μg_TAFP_/g_F_). These two levels of inclusion were chosen to mimic the cases of accidental contamination occurring along the food chain.

As emphasized in a recent review [24], most MS-based methods for analysing hidden traces of allergens in food matrices through the detection of the respective allergenic proteins rely on the well-established bottom-up proteomics protocol. Specifically, protein extraction from the three samples (cookie and rusk matrices: allergen-free, incurred Level 1, incurred Level 2) was followed by in vitro digestion by using trypsin as enzyme. Proteo-typic peptides were then analysed by liquid chromatography-tandem mass spectrometry.

Analyses were performed on a High-Resolution Mass Spectrometer platform. Following this, the sensitivity of the method, along with the evaluation of the effect of the processing conditions on allergens detection—allowing us to estimate the overall recovery—for each allergenic ingredient, was calculated, taking into consideration the effect of the processing the food underwent on the detection of the peptide-markers.

The present paper is based on the results of the ThRAll project, whose aim was tailored to develop a standardized and sensitive MS-MS based method for allergens quantification in complex food matrices like chocolate bars [25,26,27,28]. Milk, egg, peanut, soybean, hazelnut, and almond, known to be frequent contaminants of foods, were taken into consideration and investigated, and a total of 16 peptide markers were identified and validated in incurred foods, proving to be valuable markers for the six selected allergenic ingredients as thoroughly described elsewhere [25,26]. The challenge in this study was to assess the applicability of the method and robustness of the previously selected peptide-markers in detecting eggs, milk, soy, almond, hazelnut, and peanuts traces in bakery products produced under either soft or harsh conditions, namely cookies and rusks, and to investigate the sensitivity of the method in these two types of bakery products. Sesame was also included as additional prioritized allergen due to its widespread occurrence along the bakery production chain [15].

## 2. Materials and Methods

### 2.1. Chemicals

The following solvents and reagents were purchased from Sigma-Aldrich (Milan, Italy): acetonitrile for HPLC ≥ 99.9% CAS: 75-05-8, methanol for HPLC ≥ 99.9% CAS: 67-56-1 and Milli-Q water, formic acid ≥ 98% CAS: 64-18-6, ammonium bicarbonate (AB) CAS: 1066-33-7, hydrochloric acid 37% CAS: 7647-01-0, iodoacetamide CAS: 144-48-9, dithiothreitol CAS: 3483-12-3, and TRIS Ultra Pure Grade CAS: 77-86-1. Trypsin Gold Mass Spectrometry Grade CAS: 9002-07-7 was purchased from Promega (Milan, Italy), while cellulose acetate syringe filters, 5 μm (size 25 mm), were purchased from Sartorius Italy S.r.l. (Varedo, Monza e Brianza, Italy). Disposable desalting cartridges PD-10 were purchased from Cytiva, GE Healthcare Life Sciences (Milan, Italy), and Sep-Pak C18 VCA (C18, Particle Size: 55–105 µm, Pore Size: 125 Å, Silica, 50 mg, 1 cc) were purchased from Waters Spa (Sesto S. Giovanni, Milano, Italy).

### 2.2. Production of Cookies at Pilot-Scale Level

Cookies were produced in a food pilot plant in order to mimic real production processes. The matrix was incurred at two nominal concentration levels of cow’s milk, hen’s egg, peanut, soybean, hazelnut, almond, and sesame, namely 24 and 48 µg of total allergenic food proteins per g of food, hereafter referred to as μg_TAFP_/g_F_. An allergen-free cookie was produced as well. These two levels of inclusion were chosen as representative of real cases of accidental contamination and thus were useful to test the sensitivity of the developed method beyond the calculation of overall recovery.

In detail, cookies were produced according to an industrial-designed recipe already described in a recent study with slightly modifications [29].

Firstly, the following ingredients were mixed in two separate bowls: (a) powdered sugar and sunflower seed oil and (b) water, baking soda, and ammonium bicarbonate. Secondly, the content of both bowls was independently mixed thoroughly for 4 min to form a creamy compound (“creaming” phase) and then combined and mixed. Thirdly, flour was added, and the resulting dough was mixed thoroughly (final dough weight = approximately 5 kg).

After forming, cookies (approximately 10 g each) were baked at 180 °C for 11 min. For incurred cookies preparation, allergenic ingredients such as soy, sesame, hazelnut, peanut and almond, after being ground into a fine powder, were added to the flour and left to stir overnight for the purposes of homogeneity. On the contrary, milk and eggs, as liquids, were added during the third step of the production procedure.

**Figure 1:**
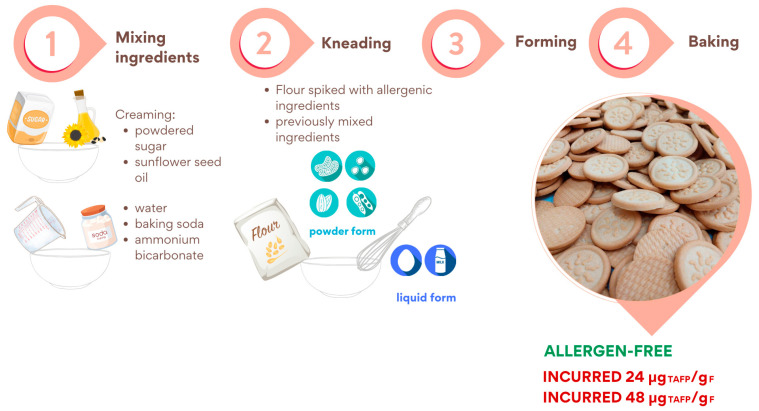
Schematic representation of the incurred and allergen-free cookies production procedure.

Figure 1 shows the cookie production steps in detail: mixing the ingredients, kneading, forming, and baking.

### 2.3. Production of Rusks at Pilot-Scale Level

Allergen-free rusks along with samples enriched with the seven allergenic ingredients under investigation (eggs, milk, soy, sesame, hazelnut, peanut, and almond) at the inclusion level of 24 and 48 μg_TAFP_/g_F_ were obtained.

The rusk-making process follows an industrial recipe already described in a recent study [30], with slight modifications, and includes the ingredients listed below: wheat flour, high oleic sunflower oil, malt, salt, yeast (*S. cerevisiae*), auxiliary agents for bakery (enzymes), and dextrose.

**Figure 2:**
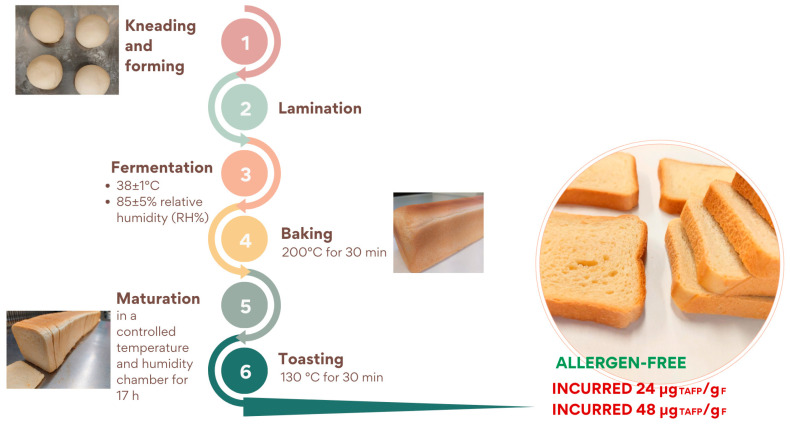
Schematic representation of rusks production at pilot-scale level.

The production process consisted of the phases summarised in Figure 2: (a) kneading of all ingredients in a planetary kneader for about 10 min; (b) formation of dough rounds and resting for 5 min at room temperature; (c) laminating step: dough was shaped to a thickness of 2.5–3.5 cm, rolled on itself, and placed in specific rectangular pans and fermented at different conditions in a leavening cell (FermaLievita Alaska, Bologna, Italy) at a 38 ± 1 °C temperature and a 85 ± 5% relative humidity (RH%); (d) baking in a pilot-scale static oven (Tagliavini, Parma, Italy) at 200 °C for 30 min; (e) maturation phase in a cell at controlled temperature and humidity for 17 h; (f) the resulting bread was sliced and toasted in a pilot-scale dynamic oven at 130 °C for 30 min (Tagliavini, Parma, Italy).

The allergen-free and incurred rusks at two levels of contamination (24 and 48 μg_TAFP_/g_F_) were prepared similarly to the detailed procedure described for the cookie.

Specifically, allergenic ingredients were added at the beginning of the production process: peanuts, almond, hazelnut, sesame, and soy powder were added to the flour, while milk and eggs were incorporated into the liquid ingredients during the kneading phase.

### 2.4. Sample Preparation Protocol Applied to Cookies and Rusks

Allergen-free cookies and rusks samples along with the two levels (24 and 48 μg_TAFP_/g_F_) incurred materials, were ground in a laboratory blender (Sterilmixer 12 6805–50 PBI International, Milan, Italy) and passed through a 1 mm sieve before being submitted to protein extraction and tryptic digestion. A sample preparation protocol described elsewhere [26,31] was used for protein extraction from cookies and rusks and the following allergens analysis. The method was slightly modified as follows: 24 mL of extraction buffer (Tris-HCl 200 mM, pH 9.2, 5 M urea) was added to 1.2 g of sample followed by 30 min stirring and 15 min of sonication in a water bath. Samples were then centrifuged at 3080 g, and the resulting supernatant was filtered on cellulose acetate filters (5 μm) and then partially purified on Size Exclusion Chromatography (SEC) with PD-10 packed columns conditioned as follows: (i) washing the storage buffer with 4 mL of Milli-Q 3 times, (ii) exchanging with 4 mL of AB 50 mM 4 times, (iii) loading of 2.5 mL of protein extracts in the cartridges and centrifugation at 1000× *g* for 2 min. Afterwards, the eluted purified protein extract was recovered.

For enzymatic digestion, a volume of 1000 μL of each sample was treated according to what described in another work [25]. The digestion mixture was then centrifuged at 5000 rpm for 10 min and the supernatant filtered through 0.45 μm RC filters before being further purified and pre-enriched on SPE SepPAk C18 columns. Before sample loading, columns were conditioned by adding 1 mL of methanol (3 times) and 1 mL of AB 50 mM (3 times), then 1000 μL of digests were loaded into the columns. After this step, the stationary phase was washed with 800 μL of Milli-Q water + 0.1% of formic acid and the purified peptides were finally eluted by adding 500 μL (3 times) of a methanol/water 90:10 solution (*v*/*v*). After elution, samples were dried under nitrogen flow and then re-suspended with 100 μL of a water/acetonitrile 95:5 (*v*/*v*) mixture containing 0.1% formic acid. Samples were finally analysed by LC-MS platform.

### 2.5. Preparation of Calibration Curves Using Spiked Samples

For allergens quantification, individual calibrations curves were obtained by adding each ingredient to a calculated amount of allergen-free cookie or rusk before sample preparation. Specifically, a stock sample was prepared at the concentration of 2000 μg_TAFP_/g_F_ (“spike stock” sample) for each allergen by adding powder of soybean, hazelnut, peanut, almond, and sesame along with milk and egg in liquid form to a defined amount of grounded allergen-free cookie or rusk. Calculations were based on the percentage protein content reported for each allergenic ingredient in the work authored by Taylor et al. (2014) [32]. The “spike stock” sample was then subjected to the extraction and purification procedure previously described. “Spike stock” purified extract was then subjected to serial dilutions with purified aliquots of allergen-free cookie sample to obtain seven calibration points in the range of 3–150 μg_TAFP_/g_F_ for each allergenic ingredient. Samples produced were then submitted to tryptic digestion and the resulting peptide mixture subsequently purified according to the protocol described above, and finally analysed in LC-HR-MS.

The study specifically focuses on the peptide markers listed in Table 1. In particular, the mentioned table reports the allergenic ingredients along with the respective marker peptides and the corresponding proteins they belong to. All the listed markers (except for sesame) were validated in earlier studies as already mentioned in the introduction [25,31,33]. For sesame peptides, ad hoc analysis were experimentally carried out in our lab in order to find the best candidate markers, also based on the information available in the literature [34,35].

The peak areas calculated for each peptide by integration of precursor ions were then correlated with the total protein of allergenic food expressed μg_TAFP_/g_F_ for the construction of the calibration lines. The values of Limit of Detection (LOD) and Limit of Quantification (LOQ), calculated as 3 and 10 times the ratio between the standard deviation on the equation intercept and its slope, are reported in the results section and led to the selection of the best-performing quantifier peptides.

### 2.6. HPLC-ESI-MS Instrumental Settings

Analyses were performed on a LC/MS platform including an UltiMate 3000 liquid chromatograph (Thermo Fisher Scientific, Waltham, MA, USA) coupled to a quadrupole-Orbitrap high-resolution hybrid mass spectrometer (Q-Exactive, Thermo Fisher Scientific, Waltham, MA, USA). The mixture of tryptic peptides was separated on an Aeris Peptide column (150 × 2.1 mm, packed with 3.6 µm particles and characterised by a XB-C18 stationary phase), purchased from Phenomenex (Castel Maggiore, Bologna, Italy).

A binary elution gradient based on the following solvents: water + 0.1% formic acid (Solvent A) and acetonitrile + 0.1% formic acid (Solvent B), operated at a flow of 200 µL/min, was adopted for the chromatographic separation. The gradient used was as follows: 0 to 35 min (10 to 35% B), 35 to 36 min (35 to 90% B), 36 to 46 min isocratic at 90% B, 46 to 47 min return to 10% of B and isocratic for the next 20 min. The injection volume was set as 20 μL. Column temperature was maintained at 30 °C throughout the chromatographic run.

As for mass spectrometry conditions, analyses were performed in positive polarity activating the targeted-Selected Ion Monitoring with data-dependent fragmentation (t-SIM/dd2) acquisition mode, which allows a highly reliable peptide identification based on the accurate detection of both the peptide precursor and the relevant MS/MS fragmentation pattern. This mode activates the potential of the quadrupole mass analyser upstream of the orbitrap analyser to perform a target analysis of the selected peptides.

The following parameters were set: time-scheduled inclusion list with 2 min wide window centred on the average retention time (t_R_), *SIM event*: microscan 1, resolution 35 k, AGC target 5 e^5^, maximum injection time 100 ms, loop count 8, isolation window 2.0 *m*/*z*, isolation offset 0.4 *m*/*z*; *dd-MS*^2^ *event*: microscan 1, resolution 17.5 k, AGC target 2 e^5^, maximum injection time 100 ms, loop count 10, isolation window 2.0 *m*/*z* units, offset 0.4 *m*/*z*, stepped collision energy (CE) 27–30 eV; *dd settings*: minimum AGC target 5.00 e^2^, intensity threshold: 5.00 e^2^, charge exclusion 1, >4, peptide match preferred, exclude isotopes on, dynamic exclusion 15 s.

The ESI source parameters set for both acquisition modes were spray voltage at 3.5 kV, capillary temperature 320 °C, sheath gas and auxiliary gas flow rates at 40 and 10 arbitrary units, and S-lens a 60.

## 3. Results and Discussion

### 3.1. Evaluation of Method Sensitivity, Method Precision and Processing Effect on Allergens Detection in Cookie

#### 3.1.1. Sensitivity

As previously described in Section 2.5, a calibration curve with seven inclusion levels spanning the range of 3–150 μg_TAFP_/g_F_ was prepared. After chromatographic separation of the peptide mixture, the respective MS spectra obtained were processed by the open-source software Skyline (https://skyline.ms/project/home/software/Skyline/begin.view, accessed on 9 September 2024) in order to calculate the areas underlying the chromatographic peaks for each allergenic ingredient after filtration of the Total Ion Current (TIC) produced by LC MS analysis on the accurate masses of each peptide (Extracted Ion Chromatogram XIC). The relevant parameters obtained for the calibration curve along with the specific parameters calculated for each peptide marker are reported in Table 2. The values of LODs and LOQs were calculated as described above. For each allergen, the most sensitive peptides potentially eligible as quantitative markers are marked in green in Table 2.

A good correlation of the regression line was obtained for all analysed peptides, showing R^2^ values comprised in the range 0.98–1.

As shown in Table 2, LOD values strictly depend on the allergenic protein. Values ≤ 3 μg_TAFP_/g_F_ were calculated for all milk peptides except FFV and for peptides ATA (egg), SPD and TAN (peanut), ADI (hazelnut), VFD (soy), TEE and ADI (almond), and SPL (sesame), while LOD values between 3 and 10 μg_TAFP_/g_F_ were displayed for peptides FFV (milk), NIG and GGL (egg), ALP (hazelnut), VLI (soy), and AFD (sesame). Similarly to what was seen for LOD, LOQs values also showed a certain variability among the peptide markers for the different allergenic ingredients, with limits of quantification ranging from 1.8 to 30 μg_TAFP_/g_F_.

The peptides showing the best instrumental performance in terms of LOD and LOQ were selected as quantifiers while the others were used as qualifiers. Selected quantifier peptides are marked in green in Table 2 and were NAV (casein-derived) and VVL (whey-derived) for milk allergens, ATA (yolk-derived) and ISQ (egg white-derived) for egg, TAN for peanut, ADI for hazelnut, VFD for soy, TEE for almond, and SPL for sesame. As a result, the calculated LODs were in the range 0.5–3 by monitoring the quantifier peptides.

#### 3.1.2. Method Precision

Cookie was chosen as food model to be further investigated for method precision as it is considered a challenging matrix very rich in proteins. The intra-day and inter-day precision of the method were assessed in this matrix within the same laboratory by analysing the samples incurred at the level of 48 μg_TAFP_/g_F._

Notably, three independent samples were prepared within the same day and on different days, covering a period of three days. Analysis was carried out by the same operator on the same instrument, with three technical replicates each. The intra-day coefficient of variation (CV%) was satisfactory and always lower than 30% for all quantitative markers monitored at the inclusion level tested (Table 3).

#### 3.1.3. Evaluation of the Processing Effect on Overall Allergen Recovery in Incurred Cookies

To estimate the overall allergens recovery upon the processing applied, samples prepared at pilot scale were analysed on the hybrid quadrupole-orbitrap platform. Specifically, the allergenic ingredients were added during the dough preparation to achieve two levels of contamination, 24 and 48 μg_TAFP_/g_F_. Each level was produced in duplicate and subjected to extraction, purification, and enzymatic digestion as previously outlined before undertaking LC-MS analysis.

The areas under the chromatographic peaks for each peptide, obtained by processing the MS spectra via Skyline software (version 24.1), were finally interpolated with a linear equation to build the calibration curves of each individual peptide-marker.

The ratio between the allergen amount calculated at the experimental level and the theoretical one, at both concentrations of 24 and 48 μg_TAFP_/g_F_, allowed the estimation of the overall recovery through each quantifier peptide tracing for the respective allergenic ingredient.

**Figure 3:**
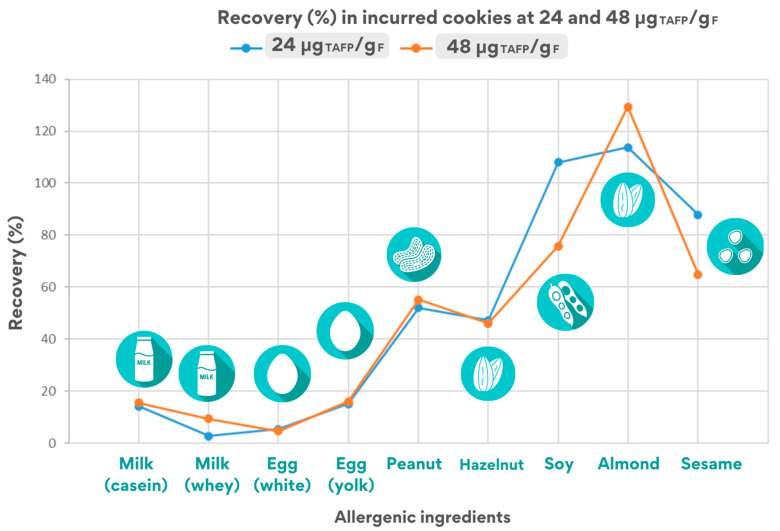
Estimated recovery rates for the quantifier peptide for each allergenic ingredient in the cookie incurred at 24 and 48 μg_TAFP_/g_F_.

Figure 3 shows a summary graph of the percentage recovery calculated for cookies incurred at 24 and 48 μg_TAFP_/g_F_.

As shown in Figure 3, the overall recovery values obtained for both levels of inclusion in cookies appear to be comparable and with the same trend, except for few specific cases, emphasizing that the same physico-chemical phenomena occur at the protein moiety independently of the inclusion level chosen.

A significant difference was found in the values calculated for the two inclusion levels analysed, reporting in the case of soy recoveries > 100% in the 24 μg_TAFP_/g_F_ incurred cookie and recoveries of 78% in the 48 μg_TAFP_/g_F_ cookie by monitoring the VFD peptide. Similar results were obtained by using the soy-qualifier VLI peptide for quantification, which nevertheless showed good calibration parameters.

Summarising, the processing applied to foods appeared to have different effect on final allergen detection as follows: (i) a very mild or negligible effect on allergens such as soy, almond, and sesame, showing a fairly stable behaviour (stability always better than 65%); (ii) a stronger effect on the detection of peanuts and hazelnuts, for which the peak areas of the corresponding marker almost halved after processing (stability around 50%); (iii) a marked effect on the detection of milk and egg, where the final amount still detectable in the processed food lay in the range of 5–15% of the original content.

Recovery values could, in general, offer some preliminary information on the effect of cookie production on the extractability/structural modifications of allergenic proteins, which could impair their final solubility with consequent influence on peptides detection. Focusing on the overall recovery values calculated for the seven allergens (considering the processing effect) in the 48 μg_TAFP_/g_F_ cookie (Figure 3), low recovery values was observed for milk ingredients (approximately 3–15%) and egg (5–16% depending on the peptide). On the contrary, higher values (from 45 to 55%) were calculated for hazelnuts and peanuts, and percentages > 60% were calculated for sesame and almond. These results suggest a high susceptibility of milk and egg proteins to cookie processing despite the resistant behaviour displayed by other allergens. As a matter of fact, it must be underlined that milk and egg were the only ingredients added in liquid form, whereas the other allergenic ingredients were added as powder during cookies production. The physical form of the incurred ingredient likely plays an important role in its stability over the processing applied. Changes in the chemical or structural forms might expose allergenic proteins prone to modifications or degradation phenomena in the food matrix in which they are entrapped. The high water content of liquid milk and fresh egg may have enhanced the alterative/degradative effect of cookie production on the major allergenic proteins of milk and egg, such as caseins and lactoglobulin in milk and ovalbumin and vitellogenin in egg, affecting the detection of relevant markers.

In conclusion, the method proved to be satisfactorily applied for allergen quantification in bakery products, tracing the presence of milk, egg, soy, almond, hazelnut, peanut, and sesame allergens in cookies, reaching LODs in the range of 1–3 μg_TAFP_/g_F_, and confirming its reliability and robustness in this complex and highly processed food matrix. The encouraging results obtained push towards a deeper investigation to be carried out in the future, looking into the detection capabilities and stability assessment of the selected peptide markers when analysing other processed foods.

### 3.2. Evaluation of Method Sensitivity and Processing Effect on Allergens Detection in Rusks

#### 3.2.1. Sensitivity Evaluation

Similarly to what was performed for the cookie matrix, spiked calibration curves for each peptide monitored referred to the different allergenic ingredients were built up in the rusk food matrix (see 2.5 paragraph).

In particular, a “spike-stock rusk” sample was prepared at 2000 μg_TAFP_/g_F_ and subjected to the sample preparation protocol. The partially purified extract was then diluted with the allergen-free rusk extract to obtain seven concentration levels for the construction of the different calibration lines (range 3–150 μg_TAFP_/g_F_ for each allergenic ingredient). Extracts referred to each calibration point were then digested and cleaned up according to the protocol described above and finally analysed in LC-HR MS by running the instruments in T-SIM/dda acquisition mode.

The relevant parameters calculated for each peptide are detailed in Table 4.

The peak areas of most of peptides precursor ions were recorded and used for quantification purposes, except for the peptides ATA and TAN, belonging to egg yolk and peanut, respectively. In both cases, the presence of coeluting isobaric species prevented the accurate extraction of the precursor ion current. For the TAN peptide, this issue was overcome by considering the information obtained from the most sensitive transition, namely the y_6_^+^ transition (628.3721 → 741.4981) that was used as the reporter ion for the TAN peptide throughout the entire dataset.

By contrast, for the ATA peptide, the presence of interfering peaks and the lower abundance of the source protein (vitellogenin 1) in the egg material used for incurring the food impaired the overall detection, with a very weak intensity of the MS/MS fragmentation pattern as well.

For all peptides, good linearity of the response with linear correlation coefficients always better than 0.998 was obtained.

As for LODs, values ranged from 2 to 10 μg_TAFP_/g_F_, depending on the allergenic ingredient/peptide, and were typically slightly higher than those calculated for cookies. These results could be explained by taking into account the complexity of the process the rusk undergoes for its production, which could give rise to more pronounced protein alteration and/or protein binding with matrix components compared to that obtained with cookies, with direct consequences on the detection sensitivity.

As reported in Table 4, challenging LODs were achieved for FFV, NAV (milk), TAN (peanut), VLV (milk), NIG and ISQ (egg), SPD (peanut), VFD (soy), ADI (almond), and AFD and SPL (sesame). Higher LODs values, ranging from 6 to 10 μg_TAFP_/g_F_**,** were instead obtained for the remaining peptides, namely IDA (milk), GGL (egg), ADI and ALP (hazelnut), VLI (SOY), and TEE (almond).

Similarly to what was observed for the LODs, the LOQs values also showed a certain variability among the peptides for the different allergenic ingredients, with limits ranging from 7 to 30 μg_TAFP_/g_F_.

For quantitation purposes, peptides showing the highest sensitivity and best LOD and LOQ were selected as quantifier peptides, while the remaining peptides were used as qualifier peptides. The selected quantifier peptides, marked in green in Table 4, included NAV (casein-derived) and VVL (whey-derived) for milk allergen, NIG (yolk-derived) and ISQ (egg white-derived) for egg, TAN for peanut, ADI for hazelnut, VFD for soy, ADI for almond, and SPL for sesame.

In accordance with these results, the same quantifier peptides could be used for monitoring the presence of milk casein derivates (NAV), whey derivates (VLV), egg white (ISQ), peanut (TAN), hazelnut (ADI), soy (VFD), and sesame (SPL) independently in cookies and rusks, proving to be the most sensitive and reliable peptide markers, able to trace any potential risk of cross-contamination. The LOD values achieved for the monitored allergens were also comparable for both type of bakery food products. On the other hand, it is worth noting that some issues were encountered for some peptides, such as ATA (egg yolk). Indeed, peak integration of the corresponding precursor ion was not possible in the rusk matrix due to the presence of an interfering compound. This led to the exclusion of this peptide from further discussions.

#### 3.2.2. Evaluation of the Processing Effect on Overall Allergen Recovery in Incurred Rusks

**Figure 4:**
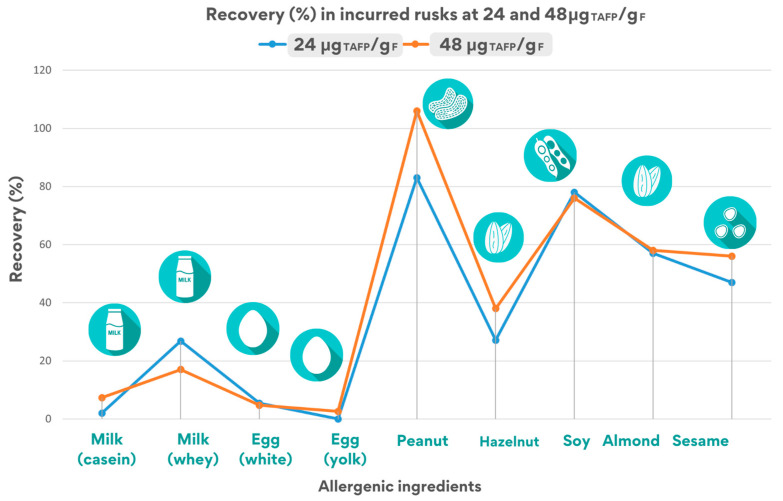
Estimated recovery rates for the quantifier peptide for each allergenic ingredient in the rusk incurred at 24 and 48 μg_TAFP_/g_F_.

The analysis of the incurred rusks produced at two concentration levels (24 and 48 μg_TAFP_/g_F_) was performed according to the described protocol, and the total allergenic food protein content was determined by interpolation of the ‘spiked’ calibration curves (Figure 4). The chromatographic peak areas for each peptide were processed via Skyline software. The recovery of each quantifier peptide for allergenic ingredients was estimated by comparing the experimentally calculated allergen amounts to the theoretical values (24 and 48 μg_TAFP_/g_F_).

For these samples, two independent biological replicates (independent extraction) were prepared, each analysed in a technical duplicate.

Figure 4 summarizes the recovery percentages of each quantifier peptide for allergenic ingredients in rusks, tested at 24 and 48 μg_TAFP_/g_F_.

As illustrated in Figure 4, the recovery values calculated in incurred rusks at both inclusion levels (24 and 48 μg_TAFP_/g_F_) are quite similar, demonstrating the robustness of the analytical method.

In the 24 μg_TAFP_/g_F_ rusk sample, at least two peptides were detected for each allergenic food, although with an overall recovery varying from 2% to 90% due to the different processing effect on the specific allergenic protein. In particular, recovery was found to be acceptable (i.e., in the range 50–150%) for at least one marker for peanut (83 ± 19%), soy (91 ± 21%), almond (57 ± 8%), and sesame (49 ± 6%), while it was found to be lower for egg (33 ± 1%), milk (26 ± 1%), and hazelnut (37 ± 7%).

A similar trend was observed for the inclusion level 48 μg_TAFP_/g_F_, where all peptides were detected, including the ISQ peptide tracing for egg white that was monitored for egg. Similar recovery values were obtained for peanuts (106 ± 10%), soy (91 ± 6%), almonds (58 ± 11%), sesame (56 ± 7%), and hazelnut (44 ± 2%), while a remarkable lower recovery was found for egg (21 ± 1%), and milk (17 ± 12%), highlighting the heavy effect of the processing applied to these allergens, probably because they were present in a liquid form.

The calculation of the overall recovery (considering the processing effect) could also provide some preliminary insights into how rusk production may affect protein extractability or the structural integrity of allergenic proteins, thus altering their solubility and influencing peptides detection.

As described above, as for rusks incurred at 48 μg_TAFP_/g_F_ (Figure 4), a high processing effect was displayed for milk ingredients (approximately in the range 7–17%) and egg (between 2–21%, depending on the specific peptide). Conversely, a higher stability (and hence final recovery) was noticed for almond and sesame (ranging from 46–58%), while percentages exceeding 70% were calculated for peanut and soy.

As already mentioned before, the low stability of milk- and egg-derived peptides underpin the hypothesis that when used as liquids, these allergenic ingredients show more susceptibility to heat treatments. On the contrary, for the other allergenic ingredients, the selected markers appear to be very robust, also confirming them to be valid makers to trace the presence of these allergenic ingredients in rusks despite the harsh conditions applied for their production, including fermentation, baking (200 °C for 30 min), and toasting (130 °C for 30 min).

### 3.3. Comparison of Allergens Detection in Cookie and Rusks

In this section, a general comparison between the performance of the methods developed to detect milk, egg, soy, almond, peanut, hazelnut, and sesame in cookie and rusk is described with the aim of evaluating the robustness of the peptides selected as markers and the influence of the food matrix complexity on the method performance. In detail, the sensitivity and recovery of the methods were compared.

#### 3.3.1. Method Performance: Incurred Cookies vs. Incurred Rusks

The list of peptide markers previously selected and considered good candidates for tracing milk, egg, soy, almond, peanut, and hazelnut in chocolate bars [25] was assessed for their applicability and feasibility to trace the relative allergens in two types of bakery products—cookies and rusks—undergoing different technological treatments. They proved to successfully detect allergen traces in cookies and rusks with very challenging sensitivities, although differences in LOD and LOQ values were observed depending on the specific peptide monitored and the type of food matrix analysed. Some remarkable differences were observed in assessing peptide robustness in both products. For example, the ATA peptide was detected in cookie, whereas it presented strong matrix interferences in rusks which totally impaired its detectability. Likewise, for the TAN peptide tracing for peanut, the presence of a co-eluting isobaric species hindered the accurate extraction of the precursor ion current, and consequently data from a specific transition (y_6_^+^, 628.3721 **→** 741.4981) were considered.

**Figure 5:**
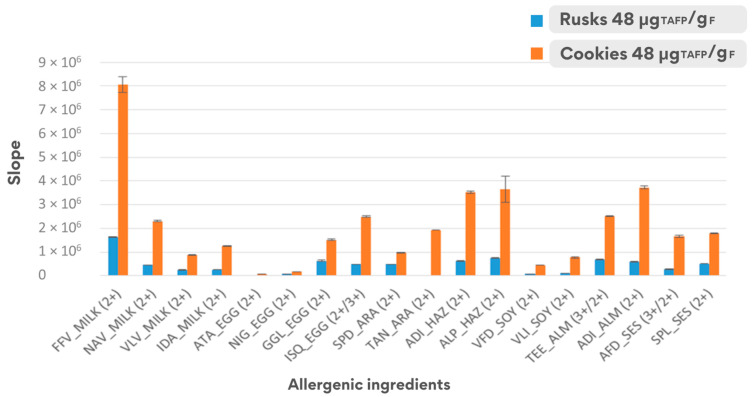
Comparison of the sensitivity (slope ± dev.st.) of the calibration curves calculated with spiked samples in the range 3–150 μg_TAFP_/g_F_ for the two matrices under investigation.

Regarding the equation parameters of the calibration regression line, a significant difference was noticed for the respective slopes. This is probably due to the different matrix effect when analysing both food matrices, and the higher slope confirmed the greater sensitivity of the analytical method for allergen detection when applied to the cookie matrix (see Figure 5).

#### 3.3.2. Evaluation of the Processing Condition on Allergens Detection

A comparison between the two analysed matrices, cookies and rusks, reveals notable differences in the overall recovery rates. These variations can be attributed to the distinct complexity of the matrices as well as the specific processing conditions applied during their production, which impact protein extractability and peptide quantification.

**Figure 6:**
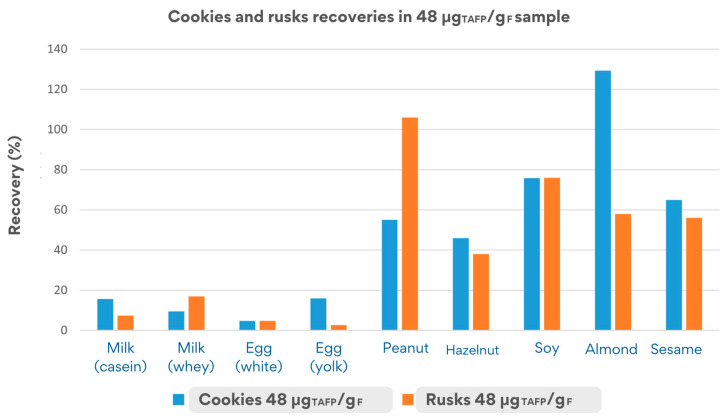
Comparison of recovery values obtained in cookies and rusks incurred at 48 μg_TAFP_/g_F_.

The recovery values depicted in Figure 6 were obtained by monitoring the quantifier peptides in both matrices, cookies and rusks.

The cookie matrix, less complex than rusks (since the fermentation step is missing for cookies), showed acceptable overall recovery rates (50–150%) for at least one peptide per allergens for peanut, almond, hazelnut, soy, and sesame. In stark contrast, milk and egg allergens showed very low recovery rates (3–16%), likely due to the high susceptibility of their parent proteins (e.g., caseins, ovalbumin) to processing-dependent degradation. The liquid state of the allergens added to the matrix likely influenced protein behaviour during production, resulting in up to a 90% reduction in their initial concentration within the matrix.

The rusk matrix, subjected to more intense processing (e.g., baking and toasting), showed similar low recovery rates for milk and egg proteins (2–21%), confirming their structural instability in liquid form. Conversely, acceptable recovery rates were observed for peanut, almond, soy, and sesame, with values exceeding 70% for peanuts and soy and moderate recovery (46–58%) for almond and sesame, highlighting very promising results, even in the case of the processed matrix.

In general, the cookie matrix showed a higher stability for many allergens, such as almonds, milk, and egg, compared to rusks, likely due to the less harsh conditions of the processing applied during production.

## 4. Conclusions

The primary challenge of this study was to assess the validity of the allergen markers identified in the ThRAll project in baked food products. The list of peptide markers previously identified was assessed in other baked goods such as cookies and rusks, known to be complex and processed food products.

The results gathered highlight the robustness of the seven peptide markers identified tracing for milk, egg, peanut, hazelnut, soy, almond, and sesame (with the addition of other two specific peptide markers tracing for yolk egg and whey protein derivatives) that were successfully validated across various goods, thus broadening their applicability to diverse food systems. This represents a noteworthy outcome when considering the routine analysis conducted in the production facilities handling a wide array of food products. The ability to reliably detect allergenic ingredients, even in highly complex matrices subjected to multiple processing steps, would substantially improve the flexibility and efficiency of quality control systems under these settings. Such methods could streamline allergen monitoring across various product lines, ensuring consumer safety while maintaining the versatility required by manufacturers.

In conclusion, the multi-target MS based method developed through a bottom-up approach could offer a viable solution for managing hidden allergens, even in highly processed baked products such as rusks, which may also be very useful in future for PAL implementation integrated with allergen control plans. The goal of ensuring the traceability of allergenic components post-processing has been successfully achieved. In future, more investigation will be carried out to fully validate the whole analytical method in both food products, also encompassing other hard-to-analyse matrices, and to deepen the knowledge on how the type of processing might affect chemical modifications of the whole protein moiety and to what extent it can alter allergen detection through their proteotypic peptide markers.

## Figures and Tables

**Table 1:** Marker peptides selected for each of the seven allergenic ingredients and their corresponding proteins.

Allergenic Ingredients	Protein	Mass [*m*/*z*]	Charge	Peptide Markers
Milk—casein	Bos d 9/αS1-casein	692.8686	2+	FFVAPFPEVFGK
	Bos d 10/αS2-casein	598.3433	2+	NAVPITPTLNR
Milk—whey	Bos d 5/β-lactoglobulin	533.295	2+	VLVLDTDYK
	Bos d 5/β-lactoglobulin	458.7404	2+	IDALNENK
Egg—white	Gal d 2/Ovalbumin	637.3486	2+	GGLEPINFQTAADQAR
	Gal d 2/Ovalbumin	479.7638	2+	ISQAVHAAHAEINEAGR
Egg—yolk	Gal d 6/YPG 42	844.4236	2+	ATAVSLLEWQR
	Vitellogenin 2	479.7638	2+	NIGELGVEK
Soy	Gly m 6/Glycine	695.3541	2+	VFDGELQEGR
	Gly m 6/Glycine	628.3721	2+	VLIVPQNFVVAAR
Almond	Pru du 6/Prunin	576.2882	2+	TEENAFINTLAGR
	Pru du 6/Prunin	815.4334	2+	ADIFSPR
Peanut	Ara h 3	575.2804	2+	SPDIYNPQAGSLK
	Ara h 3	713.4325	2+	TANDLNLLILR
Hazelnut	Cor a 9	576.2882	2+	ADIYTEQVGR
	Cor a 9	403.2138	2+	ALPDDVLANAFQISR
Sesame	11S globulin seed storage protein	1075.5419	2+	AFDAELLSEAFNVPQETIR
	11S globulin seed storage protein	582.3246	2+	SPLAGYTSVIR

**Table 2:** Parameters related to the calibration curves of the seven allergenic ingredients with calculation of LOD and LOQ obtained in TSIM/dda acquisition mode in the cookie matrix. For each allergen, the most sensitive peptides to be used as quantitative marker peptides are shown in green.

Allergenic Ingredient	Peptide Code	Precursor (*m*/*z*)	Calibration Lines Parameters (y = bx + a)					LOD (3 × S_a_/b) [μg_TAFP_/g_F_]	LOQ (10 × S_a_/b) [μg_TAFP_/g_F_]
			R2	b	Sb	A	Sa		
Milk—casein	FFV_MILK (2+)	692.8686	0.99	8,063,229	333,587	+12,043,499	26,972,746	10	30
	NAV_MILK (2+)	598.3433	1.00	2,301,911	32,058	+3,616,827	2,186,757	3	10
Milk—whey	VLV_MILK (2+)	533.295	1.00	870,501	11,718	+2,006,089	799,345	3	9
	IDA_MILK (2+)	458.7404	1.00	1,245,617	14,907	−400,015	1,062,620	3	9
Egg—yolk	ATA_EGG (2+)	637.3486	1.00	68,454	954	+595,817	48,125	2	7
	NIG_EGG (2+)	479.7638	1.00	172,260	5433	+840,757	388,432	7	20
Egg—white	GGL_EGG (2+)	844.4236	1.00	1,519,433	34,502	+7,552,990	2,252,328	4	15
	ISQ_EGG (2+/3+)	Sum 2+/3+	1.00	2,500,388	36,970	+2,218,510	2,613,807	3	10
Peanut	SPD_ARA (2+)	695.3541	1.00	970,739	11,730	+4,342,061	800,142	2	8
	TAN_ARA (2+)	628.3721 *	1.00	1,931,608	11,996	−465,453	818,276	1.3	4
Hazelnut	ADI_HAZ (2+)	576.2882	1.00	3,520,637	46,141	+11,780,865	3,012,089	3	9
	ALP_HAZ (2+)	815.4334	0.98	3,649,008	543,276	+7,069,313	4,312,117	4	12
Soy	VFD_SOY (2+)	575.2804	1.00	447,189	1163	−2492	79,352	0.5	1.8
	VLI_SOY (2+)	713.4325	1.00	770,057	22,940	+1,256,261	1,639,300	6	20
Almond	TEE_ALM (3+/2+)	Sum 2+/3+	1.00	2,511,073	14,701	+5,203,756	959,708	1.2	4
	ADI_ALM (2+)	403.2138	1.00	3,721,816	56,219	+1,498,319	3,974,720	3	11
Sesame	AFD_SES (3+/2+)	Sum 2+/3+	1.00	1,664,504	60,092	−6,259,435	4,099,057	7	20
	SPL_SES (2+)	582.3246	1.00	1,785,655	13,804	+2,047,382	975,951	1.6	5

* For this peptide, the calibration line was constructed on the transition y^6+^ 628.3721 **→** 741.4981 acquired in MS/MS.

**Table 3:** Evaluation of the method repeatability and intermediate precision cookies: relative standard deviation (CV%) referred to the monitored peptide markers.

Allergens	Peptide Markers	Repeatability	Intermediate Precision
		CV%	CV%
Milk—casein	NAVPITPTLNR	4	6
Milk—whey	VLVLDTDYK	22	24
Egg—white	ISQAVHAAHAEINEAGR	24	29
Egg—yolk	ATAVSLLEWQR	29	30
Soy	VFDGELQEGR	22	27
Almond	TEENAFINTLAGR	10	17
Peanut	SPDIYNPQAGSLK	6	14
Hazelnut	ADIYTEQVGR	13	16
Sesame	SPLAGYTSVIR	12	23

**Table 4:** Parameters related to the calibration curves of the seven allergenic ingredients obtained in TSIM/dda acquisition mode in the rusk matrix. LODs and LOQs values are reported. For each allergen, the most sensitive peptides to be used as quantitative marker peptides are shown in green.

Allergenic Ingredient	Peptide Code	Precursor (*m*/*z*)	Calibration Curve Parameters (y = bx + a)						LOD (3 × S_a_/b) [μg_TAFP_/g_F_]	LOQ (10 × Sa/b) [μg_TAFP_/g_F_]
			R^2^	b	S_b_	A	S_a_	S_y/x_		
Milk—casein	FFV_MILK (2+)	692.8686	0.999	1,639,134	21,134	3,125,694	1,506,490	2,894,804	3	9
	NAV_MILK (2+)	598.3433	0.999	457,428	4261	−1,175,260	303,736	583,646	2	7
Milk—whey	VLV_MILK (2+)	533.2950	0.997	254,228	5999	723,771	427,597	821,651	5	17
	IDA_MILK (2+)	458.7404	0.994	261,872	9389	−1,700,609	669,292	1,286,082	8	30
Egg—yolk	ATA_EGG (2+)	637.3486	N.D.							
	NIG_EGG (2+)	479.7638	0.999	78,486	1265	360,343	973,50	161,412	4	12
Egg—white	GGL_EGG (2+)	844.4236	0.989	636,190	29,497	−4,679,084	2,102,621	4,040,301	10	30
	ISQ_EGG (2+/3+)	Sum 2+/3+	0.997	487,629	13,317	163,028	869,336	1,670,075	5	18
Peanut	SPD_ARA (2+)	695.3541	0.997	488,635	11,228	304,266	800,340	1,537,897	5	16
	TAN_ARA (2+) *	628.3721 *	0.999	10,344	122	−23,492	8714	16,744	3	8
Hazelnut	ADI_HAZ (2+)	576.2882	0.995	619,518	18,743	−382,098	1,336,027	2,567,248	7	20
	ALP_HAZ (2+)	815.4334	0.997	582,961	19,224	435,744	1,620,426	2,209,788	8	30
Soy	VFD_SOY (2+)	575.2804	0.999	88,016	1303	−212,650	92,865	178,445	3	11
	VLI_SOY (2+)	713.4325	0.994	111,902	3960	−131,216	282,283	542,423	8	30
Almond	TEE_ALM (3+/2+)	Sum 2+/3+	0.995	691,432	21,531	−1,351,310	1,534,830	2,949,260	7	20
	ADI_ALM (2+)	403.2138	0.999	586,755	9436	−276,476	672,627	1,292,489	3	12
Sesame	AFD_SES (3+/2+)	Sum 2+/3+	0.998	278,131	5587	−149,501	398,245	765,250	4	14
	SPL_SES (2+)	582.3246	0.998	512,940	8938	1,364,298	637,101	1,224,224	4	12

* For this peptide, the calibration line was constructed on the transition y^6+^ 628.3721 → 741.4981 acquired in MS/MS.

## Data Availability

The original contributions presented in this study are included in the article. Further inquiries can be directed to the corresponding author.
